# Androgen Receptor–Interacting Proteins in Prostate Cancer Development and Therapy Resistance

**DOI:** 10.1016/j.ajpath.2023.12.003

**Published:** 2023-12-15

**Authors:** Zoran Culig, Martin Puhr

**Affiliations:** Experimental Urology, Department of Urology, Medical University of Innsbruck, Innsbruck, Austria

## Abstract

Endocrine therapy for prostate cancer is based on the use of drugs that diminish androgen concentration and androgen receptor (AR) signaling inhibitors and is limited by the functional consequences of AR point mutations and increased expression of constitutively active receptors. Many coactivators (>280) interact with different AR regions. Most studies have determined the expression of coactivators and their effects in the presence of increasing concentrations of androgen or the antiandrogen enzalutamide. The p160 group of coactivators (SRC-1, SRC-2, and SRC-3) is highly expressed in prostate cancer and contributes to ligand-dependent activation of the receptor in models that represent therapy-sensitive and therapy-resistant cell lines. The transcriptional coactivators p300 and CREB-binding protein (CBP) are implicated in the regulation of a large number of cellular events, such as proliferation, apoptosis, migration, and invasion. AR coactivators also may predict biochemical and clinical recurrence. The AR coactivator expression, which is enhanced in enzalutamide resistance, includes growth regulating estrogen receptor binding 1 (GREB1) and GATA-binding protein 2 (GATA2). Several coactivators also activate AR-unrelated signaling pathways, such as those of insulin-like growth factors, which inhibit apoptosis in cancer cells. They are expressed in multiple models of resistance to therapy and can be targeted by various inhibitors *in vitro* and *in vivo*. The role of the glucocorticoid receptor in endocrine therapy–resistant prostate cancer has been documented previously. Specific coactivators may interact with the glucocorticoid receptor, thus contributing to therapy failure.

Medical therapy for non–organ-confined prostate cancer (PCa) is based on inhibition of ligand-induced androgen receptor (AR) activity. Historically, *in vitro* and *in vivo* experiments have been designed to identify compounds that can inhibit androgen activity. They were followed by clinical trials that resulted in the widespread use of the nonsteroidal drugs hydroxyflutamide and bicalutamide in therapy for many years. PCa endocrine therapy is not curative and tumor progression has been studied using multiple models. Current PCa research could benefit from novel cell lines and patient-derived xenografts, resulting in the detection of multiple changes in the AR structure. Mutations in the AR ligand-binding domain may lead to agonistic effects of these drugs, stimulation of tumor cell proliferation, and *in vivo* growth. Previous findings on the role of AR in advanced PCa stimulated the chemical search for novel AR signaling inhibitors such as enzalutamide, apalutamide, or darolutamide, which improved the survival of PCa patients.^1^ However, these drugs cannot cure PCa and the mechanisms involved in cancer progression have been investigated. Thus, AR mutations also have been detected in specimens from patients treated with enzalutamide.^2^ Other researchers have focused on the appearance of the constitutively active ARs that have been detected frequently in tissues obtained from patients during therapy.^3^ Several reviews have discussed in detail the issues related to point mutations and receptors activated in the absence of AR. This review addresses other aspects of AR action, particularly in drug development, that may be important in the future. Future studies should consider the clinical relevance of AR-interacting proteins and their expression in tumor tissues.

Translational research on PCa has focused on AR coactivators, a large group of proteins that interact with receptors. They may influence selective ligand binding because it is known that AR in certain situations is activated by steroids other than testosterone and dihydrotestosterone. More than 280 coactivators have been reported in the literature. An important issue related to a particular coactivator is the determination of its relative importance for a specific function in PCa and examination of its expression at different stages of the disease (Figure 1). Some coactivators target a smaller group of genes so bioinformatic analysis can help in their identification. Small-molecule inhibitors can target coactivators and their development is important for improving current therapeutic options.

**Figure 1:**
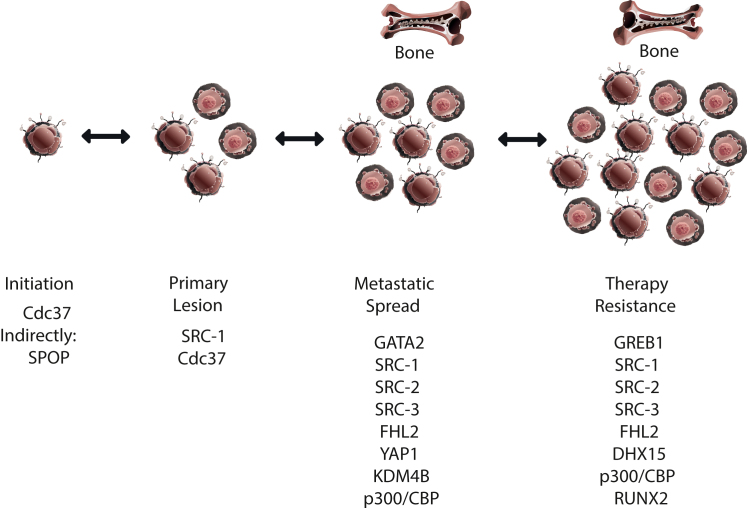
Selected cofactors with important functions at different stages of prostate carcinogenesis. Androgen receptor coactivators have distinct functions during tumor initiation and progression, as well as in therapy resistance. CBP, CREB-binding protein; Cdc37, cell division cycle 37; DHX15, DEAH-box-helicase 15; FHL2, four and a half LIM domains protein 2; GATA2, GATA-binding protein 2; GREB1, growth regulating estrogen receptor binding 1; KDM4B, lysine demethylase 4B; RUNX2, runt-related transcription factor 2; SPOP, speckle-type POZ protein; YAP1, Yes-associated protein 1.

Previous studies have discussed whether some coactivators interact exclusively with AR. Although it has been suggested that such interactions occur, most studies have analyzed the effects of cofactors that bind to multiple steroid receptors.^4^^,^^5^ Importantly, cofactor expression and function increasingly are being investigated in association with drug resistance. The AR contains a variable N-terminal region and conserved DNA- and ligand-binding domains. In general, coactivators interact with one or more receptor domains and are mentioned in this review according to this interaction, but also according to the importance of specific processes relevant to cancer progression.

## Relevance of the N-Terminal Region of the AR for Coactivation

The AR transcription activation function is present in the N-terminal region (activation function 1) and ligand-binding domain (activation function 2). The coactivators SRC-1, SRC-3, and CREB-binding protein (CBP), whose functions are discussed in detail in this article, interact with the N-terminus of AR. Therefore, approaches aimed at inhibiting AR function by blocking the N-terminus may be considered.^6^ Ralaniten (EPI-002) is an antagonist of activation function 1. Ralaniten down-regulates the transcriptional activity of AR. Interestingly, it blocks the interaction between AR and CBP, but not SRC coactivators. Under conditions in which SRC overexpression was achieved, ralaniten remained active. The N-terminal region of the AR consists of a different number of glutamine repeats. Variable length of the polyglutamine region does not affect the inhibition of transcriptional activity by EPI-002. Similarly, mutations in the N-terminal region of the receptor do not compromise the effects of the drug. In contrast to enzalutamide, ralaniten inhibits AR-V7 constitutive activity in enzalutamide-resistant cells. These results also are confirmed *in vivo*. The growth of enzalutamide-resistant LNCaP95 xenografts expressing AR-V7 is attenuated by this novel drug. Therefore, EPI-002 is a potential compound for use in clinical trials for PCa. The next-generation analog of ralaniten EPI-7170 was applied in combination with enzalutamide in PCa cells positive for AR-V7.^7^ This approach yielded a synergistic growth-inhibitory effect. A phase I study was conducted with the EPI-506 derivative, which established a safety profile, but was limited by the oral bioavailability of the drug.^8^

## The p160 Group of Coactivators in PCa

**Figure 2:**
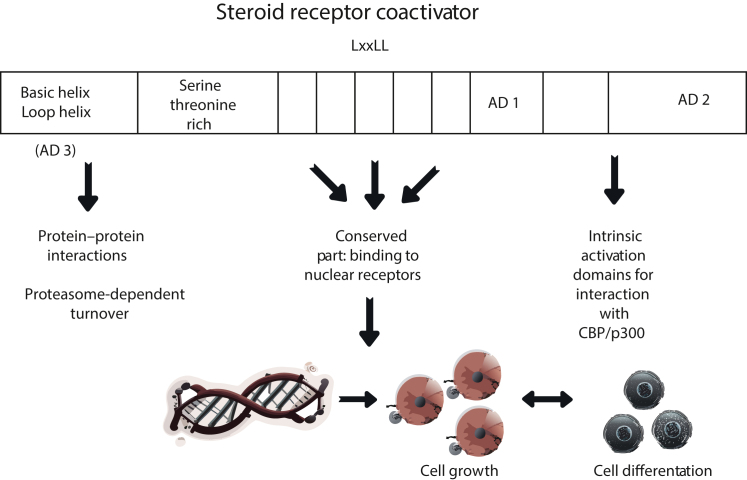
General structure of SRC coactivators. Regions responsible for protein–protein interactions, binding to nuclear receptors, and intrinsic activation domains (AD) are shown. CBP, CREB-binding protein; LxxLL, NR box motif.

The p160 coactivators of the AR, to which SRC-1, SRC-2, and SRC-3 belong [known as nuclear receptor coactivator (NCOA)1, NCOA2, and NCOA3, respectively], are functional at different stages of prostate carcinogenesis and interact strongly with the N-terminal region of the AR. These coactivators recruit histone acetyltransferases and methyltransferases to specific promoters and enhancer regions to facilitate transcription (Figure 2). They are involved in the regulation of multiple cellular processes in PCa and act via multiple mechanisms.

SRC-1 is expressed in many steroid-responsive tissues and tumors and is necessary for achieving a full hormonal response. SRC-1 is phosphorylated by mitogen-activated protein kinases to promote receptor activation. Functional SRC-1 is needed for AR activation in LNCaP cells and in their derivative C4-2, which displays high AR expression and represents a more malignant phenotype.^9^ SRC-1 coactivator is involved in the progression of PCa.^10^ Inhibition of its expression revealed reduced proliferation in two AR-positive cell lines, but no effect in PC-3 cells. This coactivator also affects cell migration and invasion. The effect of SRC-1 knockdown could be reversed by the inhibition of protein kinase D1, the expression of which was correlated inversely with that of SRC-1. Protein kinase D1 is repressed by AR. AR coactivators have been investigated in ligand-independent AR activation. Thus, SRC-1 is one of the cofactors required for AR activation by IL-6, which occurs through the N-terminal region.^11^ SRC-1 overlapping peptides interact specifically with AR and therefore could be used as a therapeutic approach in PCa. These peptides inhibited androgen-dependent PCa cells and their sublines.^12^

In general, AR coactivators can be characterized according to their regulation by androgens and are known to be androgen-induced and androgen-suppressed. SRC-2 is highly expressed after androgen ablation and promotes castration-resistant PCa.^13^ In an animal model, SRC-2 has been shown to induce early stage tumors and cancer progression. Its downstream signaling pathways include phosphatidylinositol 3-kinase/Akt and mitogen-activated protein kinases. In clinical samples, higher SRC-2 expression has been reported in recurrent tumors. SRC-2 inhibits the *SIRT3* promoter. Conversely, SRC-2 depletion enhances sirtuin 3 (SIRT3) expression. SIRT3 is regulated in the mitochondrial matrix and contains a mitochondrial processing peptide. In the same publication, it was revealed that SRC-2 coactivator enhances mitochondrial aconitase 2, which up-regulates mitochondrial citrate and facilitates *de novo* lipogenesis.^14^

SRC-3 belongs to the p160 group of proteins and regulates the steroid-receptor activity and cellular proliferation. *In vitro* observations have been confirmed *in vivo* in experiments in which inducible shRNA expression decreases tumor growth.^15^ The antiapoptotic effects of SRC-3 in PCa can be explained by its interaction with the activating protein-2 and regulation of the components of the insulin-like growth factor-1 pathway.^16^ Furthermore, it promotes cellular migration and invasion through regulation of matrix metalloproteinases.^17^ SRC-3 may contribute to PCa progression by stimulation of Akt phosphorylation.^18^ Inhibition of SRC-3 recruitment in human PCa is achieved by homeobox C8 (HOXC8).^19^ HOXC8 is a homeobox gene that thus plays an important role in the regulation of AR signaling. Interestingly, HOXC8 may promote tumor cell invasiveness when AR activity is down-regulated. More recently, the role of SRC-3 in tumor immunity has been studied.^20^ It is highly expressed in regulatory T cells and B cells. Immune cells depleted of SRC-3 generate antitumor immunity. Therapeutic approaches against SRC-1 and SRC-3 are established by the administration of gossypol to cells representing various cancers, including those of the breast and prostate.^21^ Treatment with gossypol affects cancer cells, but not normal cells. Taken together, the results in which the activity of coactivators has been inhibited indicate that their role is also important in a large number of human tumors, not only in those that are dependent on hormonal steroids. For example, SRC-3 down-regulation inhibits the growth of pancreatic ductal adenocarcinoma, confirming its role in many oncogenic signaling pathways.^22^

Consequently, targeted therapies may be developed to inhibit transcriptional coactivators in PCa. SRC-3 also has been implicated in the regulation of neuroendocrine differentiation in PCa.^23^ Neuroendocrine PCa has been the subject of many investigations, particularly because of the absence of appropriate therapies and poor prognosis. The localization of SRC proteins in PCa may be influenced by cholesterol.^24^ These studies provide an additional explanation as to how cholesterol supports androgenic signaling in PCa and nuclear translocation of receptors. Animals with hypercholesterolemia have displayed higher serum androgen concentrations. Transgenic adenocarcinoma of the mouse prostate (TRAMP) mice with global SRC-3 knockout do not develop neuroendocrine transdifferentiation.

Further studies of major interest in PCa will focus on speckle-type POZ protein (SPOP) and its interaction with SRC-3. Initially, SPOP was described as a protein that mediates transcriptional repression and interacts with components of corepressor complexes. SPOP interacts directly with SRC-3 and promotes ubiquitination and proteolysis in cancer cells. Therefore, wild-type SPOP is considered a tumor suppressor in PCa. SPOP mutations have been observed in approximately 15% of the PCa cases. The functions of SPOP may be more complex in other cancers. Consequently, the appearance of such mutations may lead to higher expression of SRC-3 and increased proliferation of cancer cells.^25^ SPOP mutations also support the activation of AR signaling and phosphatidylinositol 3-kinase/mammalian target of rapamycin, as evidenced in a mouse model of PCa.^26^ Morphologically, these mutations lead to the development of early high-grade prostate intraepithelial neoplasia, most likely in association with alterations in other oncogenes and/or tumor suppressors. By 12 months of age, most animals develop invasive and poorly differentiated cancer. pS6 and phosphorylated eiF4E-binding protein 1 (4eBP1), which are markers of mammalian target of rapamycin pathway activation, were highly expressed. SPOP mutant organoids had a higher rate of formation and irregular borders with differences in size. SPOP mutations were not observed in tumors lacking E26 transformation-specific (ETS) rearrangements. Another molecule down-regulated by SPOP in a manner similar to that of SRC-3 is the ETS-related gene (ERG) oncoprotein.^27^ As expected, clinically relevant SPOP mutants do not decrease ERG levels, thus potentiating tumor progression. SPOP mutations co-occur with deletions of the chromodomain-helicase-DNA-binding protein 1 (CHD1). Interestingly, prostate tumors with these features respond to abiraterone treatment.^28^

SPOP mutations lead to the stabilization of the tripartite motif (TRIM)24 protein, which supports cellular proliferation induced by low androgen concentrations.^29^ The TRIM24 bromodomain and AR-interacting motif are essential for growth enhancement. SPOP-mediated degradation of TRIM24 may be antagonized by TRIM28.^30^ Therefore, TRIM24 and TRIM28 interaction is important for PCa progression. Coexpression of TRIM24 and TRIM28 in PCa may result in worse clinical outcomes. TRIM33 is an oncogenic coactivator that drives prostate tumor growth by stabilizing AR from S-phase kinase associated protein 2 (Skp2)–mediated degradation.^31^ This coactivator prevents cell-cycle arrest and apoptosis.

## Multiple Functions of p300/CBP and Mediator Complex Transcriptional Coactivators in PCa

**Figure 3:**
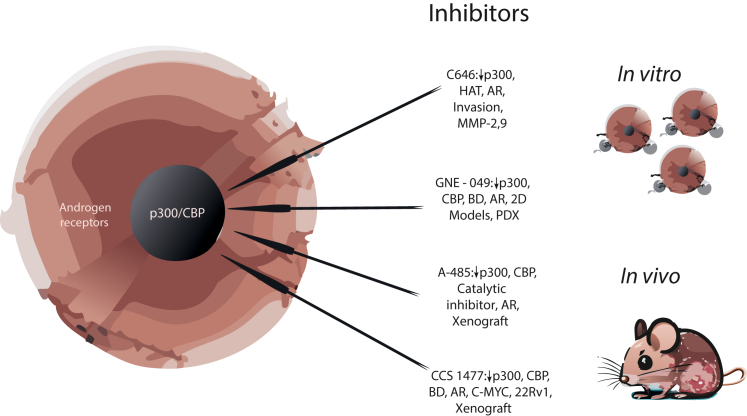
Possibilities to inhibit p300/CREB-binding protein (CBP) in prostate cancer. Compounds with different mechanisms of action have been developed and tested in cell lines and patient-derived xenografts. **Arrows** indicate p300/CBP targeting. AR, androgen receptor; BD, bromodomain; C-MYC, cellular MYC; CCS, clear cell sarcoma; GNE, glucosamine (UDP-N-acetyl)-2-epimerase/N-acetylmannosamine kinase; HAT, histone acetyltransferase; MMP, matrix metalloproteinase; PDX, patient-derived xenograft; 2D, two-dimentional; 22Rv1, human prostate carcinoma epithelial cell line.

Considerable efforts have been made to investigate the regulation of AR-mediated cellular events by the coactivators p300 and CBP.^32^^,^^33^ They modulate multiple cellular events such as proliferation, apoptosis, migration, and invasion. The regulation of AR activity by IL-6 is dependent on functional p300.^34^ These coactivators are up-regulated by androgen ablation.^35^^,^^36^ P300 binding and acetylation of AR increasingly are observed under conditions in which the tumor-suppressor phosphatase and tensin homolog (PTEN) is deleted.^37^ AR phosphorylation at serine 81 promotes p300 binding. The effects of p300 and CBP in PCa are not limited to androgen-sensitive cell lines because they enhance the migration and invasion of AR-negative PC-3 cells. Small-molecule inhibitors of p300 and CBP have been used in experimental therapies for several cancers, including PCa (Figure 3).^38^ Studies in PCa cell lines have been performed with C646. However, modifications of this compound are necessary to increase its stability. GNE-049 is a small-molecule bromodomain inhibitor that is selective for p300/CBP and has been tested *in vitro* and *in vivo*.^39^ It affects a large number of AR-regulated genes and decreases the growth of cells that are resistant to endocrine therapy. A-485 inhibits the AR program in androgen-sensitive and castration therapy–resistant cells.^38^ It can target the catalytic activity of histone acetyltransferases selectively, suggesting a possible innovation in PCa therapy. FT-6876 is a bromodomain inhibitor that causes a decrease in AR target gene expression, which is consistent with tumor growth inhibition.^40^ Treatment of castration therapy–resistant PCa with the new p300/CBP inhibitor CCS 1477 affected a considerable number of AR target genes.^41^

P300 could be considered a valid target in chemotherapy-resistant PCa.^42^ This observation is based on findings in docetaxel-resistant cells, in which the levels of p300 are higher than those in control cells. The inhibition of p300 expression affects colony formation, migration, and invasion. Docetaxel-resistant cells show higher expression of mesenchymal markers, such as N-cadherin and vimentin.^43^ AR activity is also regulated by runt-related transcription factor 2 (RUNX2).^44^ Importantly, the transcription factor SNAI2, which regulates epithelial-to-mesenchymal transition and invasion, is co-regulated by RUNX2 and AR. SNAI2 expression is correlated with RUNX2 expression. Several biological processes, such as immune responses, angiogenesis, and epithelial-to-mesenchymal transition, are regulated by transforming growth factor-β. Its intermediary molecule, suppressors of mothers against decapentaplegic homolog 3 (Smad3), promotes the expression and activity of AR.^45^ Treatment of enzalutamide-resistant cells with p300/CBP inhibitors resulted in antiandrogen effects and down-regulation of ribosomal proteins.^46^ High levels of ribosomal proteins, as a consequence of gene amplification, are observed in cells in which c-*Myc* is amplified.

In addition to p300, other coactivators may also be involved in the regulation of nonsteroidal activation of AR.^47^ NF-κB regulates several proinflammatory cytokines, such as IL-6. NF-κB is regulated by the coactivator MYST1, which interacts with sirtuin 1. Down-regulation of MYST1 activates the cleavage of poly (ADP-ribose) polymerase and caspase 3, which leads to apoptosis. In PC-3 cells, which are modified to express AR, depletion of MYST1 causes G_2_M growth arrest. Therefore, it could be concluded that MYST1 coregulates AR and NF-κB to regulate multiple cellular functions.

The elements of the mediator complex play an important role in the regulation of AR transcriptional activity. The transcriptional coactivator mediator 1 may have a particularly important role in the interaction with AR in PCa.^48^ Mediator 1 is phosphorylated at T1457 by cyclin-dependent kinase 7. In this context, down-regulation of cyclin-dependent kinase 7 leads to the inhibition of AR-mediated transcriptional amplification, thus representing a novel approach for the treatment of PCa.

## AR Coactivators and Prediction of Cancer Recurrence

Nuclear four and a half LIM domains protein 2 (FHL2), together with other oncogenic factors such as filamin, supports the progressive growth of PCa.^49^ Interaction between FHL2, AR, and filamin is particularly important for understanding the function of variant AR. Nuclear FHL2 expression was detected only in castration-resistant PCa samples. FHL2 predicts PCa recurrence risk, as evidenced by a study in which a number of other variables were analyzed.^50^ Activation of the AR by low concentrations of androgens may lead to increased expression of FHL2.^51^ Its levels are increased in tumors with a high mutation rate of p53. FHL2 has been described as a coactivator that suppresses forkhead box protein (FOX)O1 pro-apoptotic activity.^52^ Therefore, this mechanism may be important for better understanding PCa progression. A coactivator that is overexpressed in high-Gleason-score tumors and castration therapy–resistant PCa is the long noncoding RNA LINC00675.^53^ Its knockdown suppresses tumor formation and attenuates enzalutamide resistance. It modulates the interaction of the AR with mouse double minute 2 and binds to the receptor. The inhibition of LINC00675 could be combined with treatment with AR signaling inhibitors.

BAF53A is a protein member of the switch/sucrose non-fermentable (SWI/SNF) complex that regulates gene expression by gene-specific chromatin remodeling of multiple target genes.^54^ In human PCa tissues, the expression of BAF53A mRNA is increased in several publicly available databases. The depletion of BAF53A significantly decreases the growth rate of tumor cells. DEAH-box-helicase 15 (DHX15) is an RNA helicase involved in stimulating PCa progression through the up-regulation of AR.^55^ Coactivator expression is correlated with Gleason score and biochemical recurrence. It regulates AR activity through siah E3 ubiquitin protein ligase 2 (Siah2)–mediated ubiquitination. Down-regulation of DHX15 in the C4-2 derivative of LNCaP cells resulted in inhibition of xenograft growth. DHX15, similar to many other coactivators, is involved in the potentiation of AR activation at low androgen concentrations.^56^

The expression of AR also correlates with that of Yes-associated protein 1 (YAP1), which is an element of the Hippo pathway that is involved in the regulation of cellular migration.^57^ YAP1 expression is considered a prognostic factor that predicts early PCa recurrence.^58^ High YAP1 expression has been observed in therapy-resistant PCa.^59^ YAP1 and AR colocalize in hormone-naive and castration therapy–resistant PCa. Androgens have been shown to promote YAP1 nuclear abundance and activity.

## Determination of AR Coactivator Output and Its Role in the Regulation of Enzalutamide Responsiveness in PCa

The importance of studying the expression and specific action of coactivators in PCa cellular models was highlighted by Lee et al.^60^ They showed 100-fold heterogeneity in the activation of AR downstream genes in human PCa cell lines. The most important recent studies on coactivators have been conducted in PCa resistant to inhibitors of AR signaling. The medical treatment of patients with metastatic therapy–resistant disease is an unmet medical need. In addition, it is important to identify coactivators with specific functions that are not compensated by other coregulatory proteins. Cells with a higher AR output responded more strongly to low doses of androgen, which is relevant for castration resistance and reduced sensitivity to enzalutamide. It is important to analyze the consequences of the inhibition of AR coregulatory proteins in multiple models including cell lines, therapy-resistant sublines, and patient-derived xenografts. *GREB1* is up-regulated in cells with higher AR transcriptional activity. It is regulated by androgens, enhances *AR* DNA binding, and promotes the recruitment of *p300*, which is involved in several cellular processes in PCa. *GREB1* overexpression increased AR activity in a dose-dependent manner. On the other side, enzalutamide resistance can be delayed by *GREB1* knock-down *in vitro*. Inhibition of *GREB1* in enzalutamide-resistant cells restores their sensitivity to the drug. An increase in *GREB1* expression has been documented during enzalutamide treatment, which is consistent with its causative role in the development of treatment resistance. Its oncogenic role also has been investigated in ovarian cancer, in which estrogen regulation has been documented.^61^

Because of their large number and possibilities of interacting with each other, the consequences of inhibition of a coactivator of interest may not necessarily be observed. Liu et al^62^ reported 22 (of 181) clinically relevant coactivators that drive PCa progression. These coactivators were selected based on their ability to contribute to the activation of cancer-specific signaling pathways. For each androgen-regulated gene, two to four coregulators affect androgen responsiveness. Interestingly, Liu et al^62^ showed for the first time that WD repeat domain 77 (WDR77) cooperates with p53 to modulate the response to androgens. WDR77 is a component of the arginine methyltransferase protein arginine methyltransferase 5 (PRMT5)–containing complex, which modifies arginine to dimethylarginine in several spliceosomal proteins.

Another important regulator of AR signaling GATA2 (GATA-binding protein). The protein is a member of the GATA family of pioneer transcription factors and is an actionable therapeutic target.^63^ GATA2 and AR expression are correlated in PCa. When androgen levels are low, GATA2 increases AR expression, most likely by binding to the AR promoter. Consequently, GATA2 increased the levels of the AR target gene *KLK2*. It colocalizes with AR and FOXA1.^64^ FOXA1 and GATA2 strongly enhance each other's transcriptional program whereas FOXA1 is an up-stream regulator of GATA2. It is involved in regulating the transcriptional activity of full-length and variant ARs.^65^ Interestingly, He et al^65^ described a negative feedback loop because androgens cause repression of GATA2. GATA2 stimulates enzalutamide-induced transcription by facilitating AR loading at the enzalutamide-responsive gene loci.^66^ Silencing GATA2 leads to inhibition of the enzalutamide-induced genes *NR3C1* and *SLC7A11*. Consistent with these findings, the GATA2 inhibitor K7174 impaired the growth of PCa cells in response to enzalutamide. GATA2 also may be involved in PCa progression, independent of AR. This effect is achieved through up-regulation of insulin-like growth factor 2, a well-established antiapoptotic driver in cancer.^67^ Interestingly, a drug used in clinical medicine for vasodilatation (dilazep) has been identified *in silico* as a potential inhibitor of GATA2.^68^ It suppresses genes regulated by AR and c-*Myc*. In the future, clinical trials with dilazep in PCa should be conducted.

Interestingly, lysine-specific histone demethylase 1A (KDM1A; alias LSD1) acts as a transcriptional repressor by demethylating histone H3 lysine 4 and as a nuclear-receptor coactivator.^69^ It is responsible for the binding of FOXA1 chromatin to AR. Therefore, inhibition of LSD1 diminishes PCa cell growth and synergizes with AR antagonist treatment *in vivo*. Interestingly, LSD1 interaction with the AR has been studied in kidney cancer, in which it was found that the inhibitor pargyline enhances the effects of enzalutamide.^70^

## c-*Myc*, AR, and Cofactor Network

The regulation of various cellular events in PCa is largely dependent on the c-*Myc*–AR axis.^71^ Although c-*Myc* inhibits apoptosis, it stimulates glycolysis and cell-cycle progression. A complex relationship exists between AR and c-*Myc* expression in PCa. Overexpression of c-*Myc* antagonizes the expression of AR target genes.^72^ In contrast, c-*Myc* expression is up-regulated by androgenic hormones. The androgen-regulated lysine methylase KDM4B causes AR-dependent transcription of c-*Myc* mRNA.^73^ Its expression is increased in enzalutamide-resistant prostate tumors. KDM4B enzymatic activity is necessary to increase AR stability by inhibiting its ubiquitination.^74^ High KDM4B levels have been observed in high-grade PCa specimens. Inhibition of KDM4B enhanced the antagonistic effect of enzalutamide in xenografts. Thus, treatment with KDM4B inhibitors may re-establish PCa sensitivity to AR signaling inhibitors.

Increased c-*Myc* in response to androgen deprivation contributes to castration-resistant PCa, whereas decreased c-*Myc* contributes to responses to therapy with higher doses of androgen, which could be considered in patients with *AR* gene amplification or in those with DNA repair deficiency.^75^ Suppression of global AR activity is associated with the redistribution of coactivators. c-*Myc*–induced AR transcription via alteration of histone modifications at the c-*Myc* binding site within the AR gene is up-regulated by ring finger protein 8 (RNF8).^76^ RNF8 promotes the recruitment of wild-type or variant ARs to the prostate-specific antigen promoter.

## Interaction of AR Coactivators with Other Cellular Proteins

AR coactivators also may interact with other proteins. For example, the co-chaperone cell division cycle 37 (Cdc37), which is up-regulated in early PCa, interacts with vav guanine nucleotide exhange factor 3 (Vav3) to promote receptor activity and tumor growth.^77^ Disruption of the Vav3–Cdc37 interaction inhibits Vav3 enhancement of AR transcriptional activity and AR N-C interaction, which is required for activation. The Vav3 diffuse B-cell lymphoma homology domain disrupts the interaction of AR-V7 with the coactivators Src1 and Vav2, leading to decreased cellular proliferation and anchorage-dependent growth, increased apoptosis, and decreased migration.^78^ The phosphorylation of the AR is regulated by activated CDC42 kinase 1 (Ack1) tyrosine kinase. Furthermore, serum resistance–associated stem-loop–interacting RNA binding protein associates with AR and the interaction is modulated by Ack1.^79^

## AR Coactivators and Tumor Cell Metabolism

The Krebs cycle is suppressed in normal epithelial cells, but not in cancer cells. The main difference between normal and cancer cells is that in benign epithelium glucose and aspartate are involved in citrate synthesis, whereas in tumor cells there is prominent oxidative phosphorylation and lipid synthesis. This metabolic switch is mediated by AR. Oxidation of citrate and production of ATP may occur before the appearance of morphologic changes in the tissue. Metabolic substrates such as lactate are necessary to sustain malignant cell growth and may be provided by the tumor microenvironment. Lactase production is stimulated by cytokines such as IL-6 and transforming growth factor-ß.

AR coactivators are involved in regulating cancer metabolism. KDM8 (alias JMJD5) is a histone lysine demethylase/dioxygenase, which is a dual coactivator of AR and pyruvate kinase M2.^80^ Increased KDM8 expression was observed in enzalutamide-resistant cells. KDM8 is a regulator of glycolytic genes and is a target for PCa therapy. Tumor metabolism also is affected by KDM4B, which has been implicated in c-*Myc*–regulated cellular events.^81^ Its expression leads to the activation of Warburg metabolism and metabolic genes such as lactate dehydrogenase A. Androgen positively influences AMP-activated protein kinase (AMPK) signaling, which increases peroxisome proliferator-activated receptor γ coactivator-1α.^82^ This transcriptional coactivator is an important regulator of mitochondrial function and oxidative phosphorylation. It may be particularly interesting to investigate the role of AR coactivators in lipid metabolism. Several steroid receptors are considered lipid-sensing factors that affect different aspects of lipid metabolism.^83^

## Coregulators and AR-Mediated Growth Inhibition

Treatment of PCa cells with higher androgen doses leads to growth inhibition and up-regulation of cell-cycle inhibitors such as p21 and p27. Transducin-β-like–related protein 1 interacts with AR and could show both coactivator and corepressor properties. Its expression in the nucleus is higher in benign tissues than in cancerous tissues.^84^ Thus, transducin-β-like–related protein 1 is a co-activator associated with androgen-mediated growth inhibition. Similarly, ten-eleven translocation 2 (TET2) binds to AR, and its loss is associated with PCa.^85^ TET2 knockdown increases LNCaP cell proliferation, migration, and wound healing. Consistent with the functional data, decreased TET2 expression in tumor tissues is associated with reduced survival.

## Glucocorticoid Receptor in Advanced PCa and Its Role in Therapy Resistance

Other steroid receptors are expressed in the prostate epithelium and tumor microenvironment. However, functional studies on some of them have not established a relationship with tumor cell growth. In contrast, recent investigations on PCa have focused on the glucocorticoid receptor (GR). GR expression decreases in early PCa but is increased during metastatic progression.^86^ GR inhibition enhances sensitivity to enzalutamide in PCa.^87^ Thus, GR expression increases in a number of preclinical models used to study enzalutamide or abiraterone resistance.^88^ Progression-free survival is reduced in patients with higher GR expression. Other studies have shown that GR-mediated therapy resistance can be reversed by BET inhibitors that bind to bromodomains.^89^ It has been recognized that the mechanism of escape from endocrine therapy involves GR activation. Other studies have shown that GR depletion delays the progression toward castration resistance.^90^ The antiapoptotic glucocorticoid-induced kinase serum/glucocorticoid-regulated kinase-1 (SGK-1) also is involved in this process. Therefore, SGK-1 inhibition has a negative effect on PCa cell viability. Conversely, SGK-1–Flag overexpression reduces the time required for tumor initiation *in vivo.*

Consistent with these findings, docetaxel resistance could be reversed by inhibition of GR in PCa cell lines, associated with the down-regulation of genes of the *Bcl2* family.^91^ The interaction of GR with ß-catenin may be relevant to docetaxel resistance and cellular stemness.^92^ Furthermore, an increase in sensitivity to radiation therapy was reported after inhibition of GR.^93^ Mechanistically, an increase of miR-99a/-100, which may be a result of treatment with mifepristone, is required to achieve the effect of radiation therapy.

Based on laboratory findings, selective GR modulators have been developed for PCa.^94^ In contrast to mifepristone, they do not influence AR signaling. A common problem associated with classic endocrine therapy in PCa is compensatory activation of the anti-apoptotic phosphatidylinositol 3-kinase pathway. Targeting the phosphatidylinositol 3-kinase pathway with the pan-Akt inhibitor ipatasertib leads to the inhibition of GR expression and activity through cell-cycle arrest. Moreover, GR inhibition is necessary to establish sensitivity to pan-Akt inhibitors.^95^ Expression of the GR and AR signature gene *MAO-A* is found in primary tissue cultures after treatment with glucocorticoids, as well as in tissues from patients obtained after neoadjuvant chemotherapy with docetaxel or mitoxantrone. A positive correlation between *MAO-A* and pathways associated with mitochondrial activity such as oxidative phosphorylation, adipogenesis, and fatty acid metabolism has been shown. *MAO-A* can be targeted in PCa, leading to an increase in the efficacy of antiandrogen therapy.^96^

Based on these results, one can expect GR-interacting proteins to have specific functions in PCa progression. GR coactivators may decrease the half-maximal inhibitory concentration required for the induction of receptor transcriptional activity. The up-regulation of selected coactivators under conditions in which high levels of GR are measured may have implications for the development of new drugs for PCa treatment. However, the GR-cofactor interaction in PCa has not been investigated sufficiently. This is in contrast to the AR-cofactor interaction that has been studied in benign prostate epithelium, premalignant lesions, early and metastatic PCa, and therapy resistance. If critical GR partners in PCa tissue can be identified in the future, small-molecule antagonists could be used to prevent or delay therapy resistance.

## Summary and Conclusions

To improve therapeutic strategies against AR coactivators, it is crucial to focus on those proteins that are highly expressed in PCa. Furthermore, knockdown strategies are involved in important cellular functions in cancer development and progression. Pharmacologic studies have led to the identification of inhibitors of coactivator functions with improved pharmacokinetic and pharmacodynamic characteristics. Recently, several laboratories have performed *in vivo* studies to confirm the potential application of small-molecule inhibitors in clinical settings. However, clinical studies with these compounds have to be designed keeping in mind that patients should be selected carefully based on their clinical status, specific molecular characteristics of the tumor, and previous therapies.

## Disclosure Statement

None declared.

## References

[1] Mitsogianni M., Papatsoris A., Bala V.M., Issa H., Moussa M., Mitsogiannis I. (2023). An overview of hormonal directed pharmacotherapy for the treatment of prostate cancer. Expert Opin Pharmacother.

[2] Korpal M., Korn J.M., Gao X., Rakiec D.P., Ruddy D.A., Doshi S., Yuan J., Kovats S.G., Kim S., Cooke V.G., Monahan J.E., Stegmeier F., Roberts T.M., Sellers W.R., Zhou W., Zhu P. (2013). An F876L mutation in androgen receptor confers genetic and phenotypic resistance to MDV3100 (enzalutamide). Cancer Discov.

[3] Kanayama M., Lu C., Luo J., Antonarakis E.S. (2021). AR splicing variants and resistance to AR targeting agents. Cancers (Basel).

[4] Miyamoto H., Yeh S., Wilding G., Chang C. (1998). Promotion of agonist activity of antiandrogens by the androgen receptor coactivator, ARA70, in human prostate cancer DU145 cells. Proc Natl Acad Sci U S A.

[5] Alen P., Claessens F., Schoenmakers E., Swinnen J.V., Verhoeven G., Rombauts W., Peeters B. (1999). Interaction of the putative androgen receptor-specific coactivator ARA70/ELE1alpha with multiple steroid receptors and identification of an internally deleted ELE1beta isoform. Mol Endocrinol.

[6] Yang Y.C., Banuelos C.A., Mawji N.R., Wang J., Kato M., Haile S., McEwan I., Plymate S., Sadar M.D. (2016). Targeting androgen receptor activation function-1 with EPI to overcome resistance mechanisms in castration-resistant prostate cancer. Clin Cancer Res.

[7] Hirayama Y., Tam T., Jian K., Andersen R.J., Sadar M.D. (2020). Combination therapy with androgen receptor N-terminal domain antagonist EPI-7170 and enzalutamide yields synergistic activity in AR-V7-positive prostate cancer. Mol Oncol.

[8] Maurice-Dror C., Le Moigne R., Vaishampayan U., Montgomery R.B., Gordon M.S., Hong N.H., DiMascio L., Perabo F., Chi K.N. (2022). A phase 1 study to assess the safety, pharmacokinetics, and anti-tumor activity of the androgen receptor n-terminal domain inhibitor epi-506 in patients with metastatic castration-resistant prostate cancer. Invest New Drugs.

[9] Agoulnik I.U., Vaid A., Bringman W.E., Erdeme H., Frolov A., Smith C.L., Ayala G., Ittmann M.M., Weigel N.L. (2005). Role of SRC-1 in the promotion of prostate cancer cell growth and tumor progression. Cancer Res.

[10] Luef B., Handle F., Kharaishvili G., Hager M., Rainer J., Janetschek G., Hruby S., Englberger C., Bouchal J., Santer F.R., Culig Z. (2016). The AR/NCOA1 axis regulates prostate cancer migration by involvement of PRKD1. Endocr Relat Cancer.

[11] Ueda T., Mawji N.R., Bruchovsky N., Sadar M.D. (2002). Ligand-independent activation of the androgen receptor by interleukin-6 and the role of steroid receptor coactivator-1 in prostate cancer cells. J Biol Chem.

[12] Nakka M., Agoulnik I.U., Weigel N.L. (2013). Targeted disruption of the p160 coactivator interface of androgen receptor (AR) selectively inhibits AR activity in both androgen-dependent and castration-resistant AR-expressing prostate cancer. Int J Biochem Cell Biol.

[13] Qin J., Lee H.J., Lin S.C., Lanz R.B., Creighton C.J., DeMayo F.J., Tsai S.Y., Tsai M.-J. (2014). Androgen deprivation-induced NCoA2 promotes metastatic and castration-resistant prostate cancer. J Clin Invest.

[14] Sawant Dessai A., Palestino Dominguez M., Chen U.-I., Hasper J., Prechtl C., Yu C., Katsuta E., Dai T., Zhu B., Jung S.Y., Putluri N., Takabe K., Zhang X.H.-F., ÓMalley B.W., Dasgupta S. (2021). Transcriptional repression of SIRT3 potentiates mitochondrial aconitase activation to drive aggressive prostate cancer to the bone. Cancer Res.

[15] Zhou H.-J., Yan J., Luo W., Ayala G., Lin S.-H., Erdem H., Ittmann M., Tsai S., Tsai M.-J. (2005). SRC-3 is required for prostate cancer cell proliferation and survival. Cancer Res.

[16] Yan J., Yu C.-T., Ozen M., Ittmann M., Tsai S.Y., Tsai M.-J. (2006). Steroid receptor coactivator-3 and activator protein-1 coordinately regulate the transcription of components of the insulin-like growth factor/AKT signaling pathway. Cancer Res.

[17] Yan J., Erdem H., Li R., Cai Y., Ayala G., Ittmann M., Yu-Lee L.-Y., Tsai S.Y., Tsai M.-J. (2008). Steroid receptor coactivator-3/AIB1 promotes cell migration and invasiveness through focal adhesion turnover and matrix metalloproteinase expression. Cancer Res.

[18] Tien J.C.-Y., Liu Z., Liao L., Wang F., Xu Y., Wu Y.-L., Zhou N., Ittmann M., Xu J. (2013). The steroid receptor coactivator-3 is required for development of castration-resistant prostate cancer. Cancer Res.

[19] Axlund S.D., Lambert J.R., Nordeen S.K. (2010). HOXC8 inhibits androgen receptor signaling in human prostate cancer cells by inhibiting SRC-3 recruitment to direct androgen target genes. Mol Cancer Res.

[20] Han S.J., Jain P., Gilad Y., Xia Y., Sung N., Jin Park M., Dean A.M., Lanz R.B., Xu J., Dacso C.C., Lonard D.M., ÓMalley B.W. (2023). Steroid receptor coactivator 3 is a key modulator of regulator T cell-mediated tumor evasion. Proc Natl Acad Sci U S A.

[21] Wang Y., Lonard D.M., Yu Y., Chow D.-C., Palzkill T.G., O'Malley B.W. (2011). Small molecule inhibition of the steroid receptor coactivators, SRC-3 and SrC-1. Mol Endocrinol.

[22] Song X., Chen H., Zhang C., Yu Y., Chen Z., Liang H., Van Buren G., McElhany A.L., Fisher W.E., Lonard D.M., O'Malley B.W., Wang J. (2019). SRC-3 inhibition blocks tumor growth of pancreatic ductal adenocarcinoma. Cancer Lett.

[23] Tien J.C., Liao L., Liu Y., Liu Z., Lee D.K., Wang F., Xu J. (2014). The steroid receptor coactivator-3 is required for developing neuroendocrine tumor in the mouse prostate. Int J Biol Sci.

[24] Pimenta R., Camargo J.A., Candido P., Ghazarian V., Goncalves V.R., Guimares V.R., Romao P., Chiovatto C., Mioshi C.M., Dos Santos G.A., Silva I.A., Birbrair A., Srougi M., Nahas W.C., Leite K.R., Vlana N.I., Reis S.T. (2022). Cholesterol triggers nuclear co-association of androgen receptor, p160 steroid coactivators, and p300/CBP-associated factor leading to androgenic axis transactivation in castration-resistant prostate cancer. Cell Physiol Biochem.

[25] Geng C., He B., Xu L., Barbieri C.E., Eednuri V.K., Chew S.A., Zimmermann M., Bond R., Shou J., Li C., Blattner M., Lonard D.M., Demichelis F., Coarfa C., Rubin M.A., Zhou P., O'Malley B.W., Mitsiades N. (2023). Prostate cancer-associated mutations in speckle-type POZ protein (SPOP) regulate steroid receptor coactivator 3 protein turnover. Proc Natl Acad Sci U S A.

[26] Blattner M., Liu D., Robinson B.D., Huang D., Poliakov A., Gao D., Nataraj S., Leonarine L.D., Augello M.A., Sailer V., Ponnala L., Ittmann M., Chinnaiyan A.M., Sboner A., Chen Y., Rubin M.A., Barbieri C.E. (2017). SPOP mutation drives prostate tumorigenesis in vivo through coordinate regulation of PI3K/mTOR and AR signaling. Cancer Cell.

[27] Gan W., Dai X., Lunardi A., Li Z., Inuzuka H., Liu P., Varmeh S., Zhang J., Cheng L., Sun Y., Asara J.M., Beck A.H., Huang J., Pandolfi P.P., Wie W. (2015). SPOP promotes ubiquitination and degradation of the ERG oncoprotein to suppress prostate cancer progression. Mol Cell.

[28] Boysen G., Rodrigues D.N., Rescigno P., Seed G., Dolling D., Riisnaes R., Crespo M., Zafeiriou Z., Sumanasuriya S., Bianchini D., Hunt J., Moloney D., Perez-Lopez R., Tunariu N., Miranda S., Figueiredo I., Ferreira A., Christova R., Gil V., Aziz S., Bertan C., de Oliveira F.M., Atkin M., Clarke M., Goodall J., Sharp A., MacDonald T., Rubin M.A., Yuan W., Barbieri C.E., Carreira S., Mateo J., de Bono J.S. (2018). SPOP-mutated/CHD1-deleted lethal prostate cancer and abiraterone sensitivity. Clin Cancer Res.

[29] Groner A.C., Cato L., de Tribolet-Hardy J., Bernasocchi T., Janouskova H., Melchers D., Houtman R., Cato A.C.B., Tschopp P., Gu L., Corsinotti A., Zhong Q., Fankhauser G., Fritz C., Poyet C., Wagner U., Guo T., Aebersold R., Garraway L.A., Wild P.J., Theurillat J.-P., Brown M. (2016). TRIM24 is an oncogenic transcriptional activator in prostate cancer. Cancer Cell.

[30] Fong K.-W., Zhao J.C., Song B., Zheng B., Yu J. (2018). TRIM28 protects TIM24 from SPOP-mediated degradation and promotes prostate cancer progression. Nat Commun.

[31] Chen M., Lingadahalli S., Narwade N., Kei Lei K.M., Liu S., Zhao Z., Zheng Y., Lu Q., Hin Ning Tang A., Chuen Wai Poon T., Cheung E. (2022). TRIM33 drives prostate tumor growth by stabilizing androgen receptor from Skp-2 mediated degradation. EMBO Rep.

[32] Debes J.D., Sebo T.J., Lohse C.M., Murphy L.M., Haugen D.A., Tindall D.J. (2005). p300 in prostate cancer proliferation and progression. Cancer Res.

[33] Santer F.R., Höschele P.P., Oh S.J., Erb H.H., Bouchal J., Cavarretta I.T., Parson W., Myers D.J., Cole P.A., Culig Z. (2011). Inhibition of the acetyltransferases p300 and CBP reveals a targetable function for p300 in the survival and invasion pathways of prostate caner. Mol Cancer Ther.

[34] Debes J.D., Schmidt L.J., Huang H., Tindall D.J. (2002). p300 mediates androgen-independent transactivation of the androgen receptor by interleukin 6. Cancer Res.

[35] Comuzzi B., Nemes C., Schmidt S., Jasarevic Z., Lodde M., Pycha A., Bartsch G., Offner F., Culig Z., Hobisch A. (2004). The androgen receptor co-activator CBP is up-regulated following androgen withdrawal and is highly expressed in advanced prostate cancer. J Pathol.

[36] Heemers H.V., Sebo T.J., Debes J.D., Regan K.M., Raclaw K.A., Murhpy L.M., Hobisch A., Culig Z., Tindall D.J. (2007). Androgen deprivation increases p300 in prostate cancer cells. Cancer Res.

[37] Zhong J., Ding L., Bohrer L.R., Pan Y., Liu P., Zhang J., Sebo T.J., Karnes R.J., TIndall D.J., van Deursen J., Huang H. (2014). p300 acetyltransferase regulates androgen receptor degradation and pTEN-deficient prostate tumorigenesis. Cancer Res.

[38] Lasko L.M., Jakob C.G., Edalji R.P., Qiu W., Montgomery D., Digiammarino E.L. (2017). Discovery of a selective catalytic p300/CBP inhibitor that targets lineage-specific tumours. Nature.

[39] Jin L., Garcia J., Chan E., de la Cruz C., Segal E., Merchant M., Kharbanda S., Raisner R., Haverty P.M., Modrusan Z., Ly J., Choo E., Kaufman S., Beresini M.H., Romero F.A., Magnuson S., Gascoigne K.E. (2017). Therapeutic targeting of the CBP/p300 bromodomain blocks the growth of castration-resistant prostate cancer. Caner Res.

[40] Caligiuri M., Williams G.L., Castro J., Battalgine L., Wilker E., Yao L., Schiller S., Toms A., Li P., Pardo E., Graves B., Azofeifa J., Chicas A., Herbertz T., Lai M., Basken J., Wood K.W., Xu Q., Guichard S.M. (2023). FT-6876, a potent and selective inhibitor of CBP/p300, is active in preclinical models of androgen receptor-positive breast cancer. Target Oncol.

[41] Welti J., Sharp A., Brooks N., Yuan W., McNair C., Chand S.N. (2021). Targeting the p300/CBP axis in lethal prostate cancer. Cancer Discov.

[42] Gruber M., Ferrone L., Puhr M., Santer F.R., Furlan T., Eder I.E., Sampson N., Schäfer G., Handle F., Culig Z. (2020). p300 is upregulated by docetaxel and is a target in chemoresistant prostate cancer. Endocr Relat Cancer.

[43] Puhr M., Hoefer J., Schäfer G., Erb H.H., Oh S.J., Klocker H., Heidegger I., Neuwirt H., Culig Z. (2012). Epithelial-to-mesenchymal transition leads to docetaxel resistance in prostate cancer and is mediated by reduced expression of miR-200c and miR-205. Am J Pathol.

[44] Little G.H., Baniwal S.K., Adisetiyo H., Groshen S., Chinge N.-O., Kim S.Y., Khalid O., Hawes D., Jones J.O., Pinski J., Schones D.E., Frenkel B. (2014). Differential effects of RUNX2 on the androgen receptor in prostate cancer: synergistic stimulation of a gene set exemplified by SNAI2 and subsequent invasiveness. Cancer Res.

[45] Jeon H.-Y., Pornour M., Ryu H., Khadka S., Xu R., Jang J., Li D., Chen H., Hussain A., Fazli L., Gleave M., Dong X., Huang F., Wang Q., Barbieri C., Qi J. (2023). SMAD3 promotes expression and activity of the androgen receptor in prostate cancer. Nucleic Acids Res.

[46] Furlan T., Kirchmair A., Sampson N., Puhr M., Gruber M., Trajanoski Z., Santer F.R., Parson W., Handle F., Culig Z. (2021). Myc-mediated ribosomal gene expression sensitizes enzalutamide-resistant prostate cancer cells to EP300/CREBBP inhibitors. Am J Pathol.

[47] Jaganathan A., Chaurasia P., Xiao G.-Q., Philizaire M., Lv X., Yao S., Burnstein K.L., Liu D.-P., Levine A.C., Mujtaba S. (2014). Coactivator MYST1 regulates nuclear factor-kappa B and androgen receptor function during proliferation of prostate cancer cells. Mol Endocrinol.

[48] Rasool R.U., Natesan R., Deng Q., Aras S., Lai P., Effron S.S., Mitchell-Velasquez E., Posimo J.M., Carskadon S., Baca S.C., Pomerantz M.M., Siddiqui J., Schwartz L.E., Lee D.J., Palanisamy N., Narla G., Den R.B., Freedman M.I., Brady D.C., Asangani I.A. (2019). CDK7 inhibition suppresses castration-resistant prostate cancer through MED1 inactivation. Cancer Discov.

[49] McGrath M.J., Binge L.C., Sriratana A., Wang H., Robinson P.A., Pook D., Fedele C.G., Brown S., Dyson J.M., Cottle D.L., Cowling B.S., Niranjan B., Risbridger G.P., Mitchell C.A. (2013). Regulation of the transcriptional coactivator FHL2 licenses activation of the androgen receptor in castrate-resistant prostate cancer. Cancer Res.

[50] Kahl P., Gullotti L., Heukamp L.C., Wolf S., Friedrichs N., Vorreuther R., Solleder G., Bastian P.J., Ellinger J., Metzger E., Schüle R., Buettner R. (2006). Androgen receptor coactivators lysine-specific histone demethylase 1 and four and a half LIM domain protein 2 predict rick of prostate cancer recurrence. Cancer Res.

[51] Heemers H.V., Regan K.M., Dehm S.M., Tindall D.J. (2007). Androgen induction of the androgen receptor coactivator four and a half LIM domain protein-2: evidence for a role for serum response factor in prostate cancer. Cancer Res.

[52] Yang Y., Hou H., Haller E.M., Nicosia S.V., Bai W. (2005). Suppression of FOXO1 activity by FHL2 through SIRT1-mediated deacetylation. EMBO J.

[53] Yao M., Shi X., Li Y., Xiao Y., Buttler W., Huang Y., Du L., Wu T., Bian X., Shi G., Ye D., Fu G., Wang J., Ren S. (2020). LINC00675 activates androgen receptor axis signaling pathway to promote castration-resistant prostate cancer progression. Cell Death Dis.

[54] Jin M.L., Kim Y.W., Jeong K.W. (2018). BAF53A regulates androgen receptor-mediated gene expression and proliferation in LNCaP cells. Biochem Biophys Res Commun.

[55] Jing Y., Nguyen M.M., Wang D., Pascal L.E., Guo W., Xu Y., Ai J., Deng F.M., Masoodi K.Z., Yu X., Zhang J., Nelson J.B., Xia S., Wang Z. (2018). DHX15 promotes prostate cancer progression by stimulating Siah2-mediated ubiquitination of androgen receptor. Oncogene.

[56] Xu Y., Song Q., Pascal L.E., Zhong L.E., Zhong M., Zhou, Zhou J., Deng F.-M., Huang J., Wang Z. (2019). DHX15 is up-regulated in castration-resistant prostate cancer and required for androgen receptor sensitivity to low DHT concentrations. Prostate.

[57] Zhang L., Yang S., Chen X., Stauffer S., Yu F., Lele S.M., Fu K., Datta K., Palermo N., Chen Y., Dong J. (2015). The hippo pathway effector YAP regulates motility, invasion, and castration-resistant growth of prostate cancer cells. Mol Cell Biol.

[58] Marx A., Schumann A., Höflmayer D., Bady E., Hube-Magg C., Möller K., Tsourlakis M.C., Steurer S., Büscheck F., Eichenauer T., Clauditz T.S., Graefen M., Simon R., Sauter G., Izbicki J.R., Huland H., Heinzer H., Haese A., Schlomm T., Bernreuther C., Lebok P., Polonski A. (2020). Up regulation of the Hippo signalling effector YAP1 is linked to early biochemical recurrence in prostate cancer. Sci Rep.

[59] Kuser-Abali G., Alptekin A., Lewis M., Garraway I.P., Cinar B. (2015). YAP1 and AR interactions contribute to the switch from androgen-dependent to castration-resistant growth in prostate cancer. Nat Commun.

[60] Lee E., Wongvipat J., Choi D., Wang P., Sun Lee Y., Zheng D., Watson P.A., Gopalan A., Sawyers C.L. (2019). GREB1 amplifies androgen receptor output in human prostate cancer and contributes to antiandrogen resistance. Elife.

[61] Hodgkinson K., Forrest L.A., Vuong N., Garson K., Djordjevic B., Vanderhyden B.C. (2018). GREB1 is an estrogen receptor-regulated tumour promoter that is frequently expressed in ovarian cancer. Oncogene.

[62] Liu S., Kumari S., Hu Q., Senapati D., Venkadakrishnan V.B., Wang D., DePriest A.D., Schlanger S.E., Ben-Salem S., May Valenzuela M., Willard B., Mudambi S., Swetzig W.M., Das G.M., Shourideh M., Koochekpour S., Moscovita Falzarano S., Magi-Galluzzi C., Yadav N., Chen X., Lao C., Wang J., Billaud J.-N., Heemers H.V. (2017). A comprehensive analysis of coregulator recruitment, androgen receptor function and gene expression in prostate cancer. Elife.

[63] Bohm M., Locke W.J., Sutherland R.L., Kerich J.G., Henshall S.M. (2009). A role for GATA-2 in transition to an aggressive phenotype in prostate cancer through modulation of key androgen-regulated genes. Oncogene.

[64] Zhao J.C., Fong K.W., Jin H.J., Yang Y.A., Kim J., Yu J. (2016). FOXA1 acts upstream of GATA2 and AR in hormonal regulation of gene expression. Oncogene.

[65] He B., Lanz R.B., Fiskus W., Geng C., Yi P., Hartig S.M., Rajapakshe K., Shou J., Wie L., Shah S.S., Foley C., Chew S.A., Eedunuri V.K., Bedoya D.J., Feng Q., Minami T., Mitsiades C.S., Frolov A., Weigel N.L., Hilsenbeck S.G., Rosen D.G., Palzkill T., Itmann M.M., Song Y., Coarfa C., ÓMalley B.W., Mitsiades N. (2014). GATA2 facilitates steroid receptor coactivator recruitment to the androgen receptor complex. Proc Natl Acad Sci U S A.

[66] Yuan F., Hankey W., Wu D., Wang H., Somarelli J., Armstrong A.J., Huang J., Chen Z., Wang Q. (2019). Molecular determinants for enzalutamide-induced transcription in prostate cancer. Nucleic Acids Res.

[67] Vidal S.J., Rodriguez-Bravo V., Quinn S.A., Rodriguez-Barrueco R., Lujambio A., Williams E., Sun X., de la Iglesia-Vicente J., Lee A., Readhead B., Chen X., Galsky M., Esteve B., Petrylak D.P., Dudley J.T., Rabadan R., Silva J.M., Hoshida Y., Lowe S.W., Cordon-Cardo C., Domingo-Domenech J. (2015). A targetable GATA2-IGF2 axis confers aggressiveness in lethal prostate cancer. Cancer Cell.

[68] Kaochar S., Rusin A., Foley C., Rajapakshe K., Robertson M., Skapura D., Mason C., Berman De Ruiz K., Tyryshkin A.M., Deng J., Shin J.N., Fiskus W., Dong J., Huang S., Navone N.M., Davis C.M., Ehli E.A., Coarfa C., Mitsiades N. (2021). Inhibition of GATA2 in prostate cancer by a clinically available small molecule. Endocr Relat Cancer.

[69] Gao S., Chen S., Han D., Wang Z., Li M., Han W., Besschetnova A., Liu M., Zhou F., Barrett D., Luong M.P., Owiredu J., Liang Y., Ahmed M., Petricca J., Patalano S., Macoska J.A., Corey E., Chen S., Balk S.P., He H.H., Cai C. (2020). Chromatin binding of FOXA1 is promoted by LSD1-mediated demethylation in prostate cancer. Nat Genet.

[70] Lee K.-H., Kim B.-C., Jeong S.-H., Jeong C.W., Ku J.H., Kwak C., Kim H.H. (2020). Histone demethylase LSD1 regulates kidney cancer progression by modulating androgen receptor activity. Int J Mol Sci.

[71] Faskhoudi M.A., Molaei P., Sadrkhanloo M., Orouei S., Hashemi M., Bokaie S., Rashidi M., Entezari M., Zarrabi A., Hushmandi K., Mirzaei S., Gholami M.H. (2022). Molecular landscape of c-Myc signaling in prostate cancer: a roadmap to clinical translation. Pathol Res Pract.

[72] Barfeld S.J., Urbanucci A., Itkonen H.M., Fazli L., Hicks J.L., Thiede B., Rennie P.S., Yegnasubramanian S., DeMarzo A.M., Mills I.G. (2017). c-Myc antagonises the transcriptional activity of the androgen receptor in prostate cancer affecting key gene networks. EBioMedicine.

[73] Tang D.E., Dai Y., He J.-X., Lin L.-W., Leng Q.-X., Geng X.-Y., Fu D.-X., Jiang H.-W., Xu S.-H. (2020). Targeting the KDM4B-AR-c-Myc axis promotes sensitivity to androgen receptor-targeted therapy in advanced prostate cancer. J Pathol.

[74] Coffey K., Rogerson L., Ryan-Munden C., Alkharaif D., Stockley J., Heer R., Sahadevan K., O'Neill D., Jones D., Darby S., Staller P., Mantilla A., Gaughan L., Robson C.N. (2013). The lysine demethylase, KDM4B, is a key molecule in androgen receptor signalling and turnover. Nucleic Acids Res.

[75] Guo H., Wu Y., Nouri M., Spisak S., Russo J.W., Sowalsky A.G., Pomerantz M.M., Wie Z., Korthauer K., Seo J.-H., Wang L., Arai S., Freedman M.L., He H.H., Chen S., Balk S.P. (2021). Androgen receptor and MYC equilibration centralizes on developmental super-enhancer. Nat Commun.

[76] Zhou T., Wang S., Song X., Liu W., Dong F., Huo Y., Zou R., Wang C., Zhang S., Liu W., Sun G., Lin L., Zeng K., Dong X., Guo Q., Yi F., Wang Z., Li X., Jiang B., Cao B., Zhao Y. (2022). RNF8 up-regulates AR/ARV7 action to contribute to advanced prostate cancer progression. Cell Death Dis.

[77] Wu F., O'Peacock S., Rao S., Lemmon S.K., Burnstein K.L. (2013). Novel interaction between the co-chaperone and Rho GTPase exchange factor Vav3 promotes androgen receptor activity and prostate cancer growth. J Biol Chem.

[78] Magani F., ÓPeacock S., Rice M.A., Martinez M.J., Greene A.M., Magani P.S., Lyles R., Weitz J.R., Burnstein K.L. (2017). Targeting AR variant-coactivator interactions to exploit prostate cancer vulnerabilities. Mol Cancer Res.

[79] De Silva D., Zhang Z., Liu Y., Parker J.S., Xu C., Cai L., Wang G.G., Shelton Earp H., Whang Y.E. (2019). Interaction between androgen receptor and coregulator SLIRP is regulated by Ack1 tyrosine kinase and androgen. Sci Rep.

[80] Wang H.-J., Pochampalli M., Wang L.-Y., Zou J.X., Li P.-S., Hsu S.-C., Wang B.-J., Huang S.-H., Yang P., Yang J.C., Chu C.-Y., Hsieh C.-L., Sung S.-Y., Li C.-F., Tepper C.G., Ann D.K., Gao A.C., Evans C.P., Izumiya Y., Chuu C.-P., Wang W.-C., Chen H.-W., Kung H.-J. (2019). KDM8/JMJD5 as a dual coactivator of AR and PKM2 integrates AR/EZH2 network and tumor metabolism in CRPC. Oncogene.

[81] Wu M.-J., Chen C.-J., Lin T.-Y., Liu Y.-Y., Tseng L.-L., Cheng M.-L., Chuu C.-P., Tsai H.-K., Kuo W.-L., Kung H.-J., Wang W.-C. (2021). Targeting KDM4B that coactivates c-Myc-regulated metabolism to suppress tumor growth in castration-resistant prostate cancer. Theranostics.

[82] Tennakoon J.B., Shi Y., Han J.J., Tsouko E., White M.A., Burns A.R., Zhang A., Xia X., Ilkayeva O.R., Xin L., Ittmann M.M., Rick F.G., Schally A.V., Frigo D.E. (2014). Androgens regulate prostate cancer cell growth via an AMPK-PGC-1alpha-mediated metabolic switch. Oncogene.

[83] Alaynick W.A. (2008). Nuclear receptors, mitochondria and lipid metabolism. Mitochondrion.

[84] Daniels G., Li Y., Gellert L.L., Zhou A., Melamed J., Wu X., Zhang X., Zhang D., Meruelo D., Logan S.K., Basch R., Lee P. (2014). TBLR1 as an androgen receptor coactivator (AR) selectively activates AR target genes to inhibit prostate cancer growth. Endocr Relat Cancer.

[85] Nickerson M.L., Das S., Im K.M., Turan S., Berndt S.I., Li H., Lou H., Brodie S.A., Billaud J.N., Zhang T., Bouk A.J., Butcher D., Wang Z., Sun L., Misner K., Tan W., Esnakula A., Esposito D., Huang W.Y., Hoover R.N., Tucker M.A., Keller J.R., Roland J., Brown K., Anderson S.K., Moore L.E., Isaacs W.B., Chanock S.J., Yeager M., Dean M., Andresson T. (2017). TET2 binds the androgen receptor and loss is associated with prostate cancer. Oncogene.

[86] Mohler J.L., Chen Y., Hamil K., Hall S.H., Cidlowski J.A., Wilson E.M., French F.S., Sar M. (1996). Androgen and glucocorticoid receptors in the stroma and epithelium of prostatic hyperplasia and carcinoma. Clin Cancer Res.

[87] Arora V.K., Schenkein E., Murali R., Subudhi S.K., Wongvipat J., Balbas M.D., Shah N., Cai L., Efstathiou E., Logothetis C., Zheng D., Sawyers C.L. (2013). Glucocorticoid receptors confers resistance to antiandrogens by bypassing androgen receptor blockade. Cell.

[88] Puhr M., Hoefer J., Eigentler A., Ploner C., Handle F., Schaefer G., Kroon J., Leo A., Heidegger I., Eder I., Culig Z., Van der Pluijm G., Klocker H. (2018). The glucocorticoid receptor is a key player for prostate cancer cell survival and a target for improved antiandrogen therapy. Clin Cancer Res.

[89] Shah N., Wang P., Wongvipat J., Karthaus W.R., Abida W., Armenia J., Rockowitz S., Drier Y., Bernstein B.E., Long H.W., Freedman M.L., Arora V.K., Zheng D., Sawyers C. (2017). Regulation of the glucocorticoid receptor vie a BET-dependent enhancer drives antiandrogen resistance in prostate cancer. Elife.

[90] Isikbay M., Otto K., Kregel S., Kach J., Cai Y., Vander Griend D.J., Conzen S.D., Szmulewitz R.Z. (2014). Glucocorticoid receptor activity contributes to resistance to androgen-targeted therapy in prostate cancer. Horm Cancer.

[91] Kroon J., Puhr M., Buijs J.T., van der Horst G., Hemmer D.M., Marijt K.A., Hwang M.S., Masood M., Grimm S., Storm G., Metselaar J.M., Meijer O.C., Culig Z., van der Pluijm G. (2016). Glucocorticoid receptor antagonism reverts docetaxel resistance in human prostate cancer. Endocr Relat Cancer.

[92] Martinez S.R., Elix C.C., Ochoa P.T., Sanchez-Hernandez E.S., Aklashgari H.R., Ortiz-Hernandez G., Zhang L., Casiano C.A. (2023). Glucocorticoid receptor and ß-catenin interact in prostate cancer cells and their co-inhibition attenuates tumorsphere formation, stemness, and docetaxel resistance. Int J Mol Sci.

[93] Rane J.K., Erb H.H.H., Nappo G., Mann V.M., Simms M.S., Collins A.T., Visakorpi T., Maitland N.J. (2016). Inhibition of the glucocorticoid receptor results in an enhanced miR-99a/100-mediated radiation response in stem-like cells from human prostate cancers. Oncotarget.

[94] Kach J., Long T.M., Selman P., Tonsing-Carter E.Y., Bacalao M.A., Lastra R.R., de Wet L., Comiskey S., Gillard M., VanOpstall C., West D.C., Chan W.-C., Vander Griend D., Conzen S.D., Szmulewitz R.Z. (2017). Selective glucocorticoid receptor modulators (SGRM) delay castrate-resistant prostate cancer growth. Mol Cancer Ther.

[95] Adelaiye-Ogala R., Gryder B.E., Minh Nguyen Y.T., Nell Alilin A., Grayson A.R., Bajwa W., Jansson K.H., Beshiri M.L., Agarwal S., Rodriguez-Nieves J.A., Capaldo B., Kelly K., VanderWeele D.J. (2020). Targeting the PI3K/AKT pathway overcomes enzalutamide resistance by inhibiting induction of the glucocorticoid receptor. Mol Cancer Ther.

[96] Puhr M., Eigentler A., Handle F., Hackl H., Ploner C., Heidegger I., Schaefer G., Brandt M.P., Hoefer J., Van der Pluijm G., Klocker H. (2021). Targeting the glucocorticoid receptor signature gene Mono Amino Oxidase-A enhances the efficacy of chemo- and anti-androgen therapy in advanced prostate cancer. Oncogene.

